# Acute binocular diplopia: peripheral or central?

**DOI:** 10.1007/s00415-020-10088-y

**Published:** 2020-08-14

**Authors:** Olympia Kremmyda, Claudia Frenzel, Katharina Hüfner, Nicolina Goldschagg, Christian Brem, Jennifer Linn, Michael Strupp

**Affiliations:** 1 Department of Neurology and German Center for Vertigo and Balance Disorders, Ludwig Maximilian University, Marchioninistr. 15, 81377 Munich, Germany; 2 Department of Psychiatry, Psychotherapy and Psychosomatics, University Hospital of Psychiatry II, Medical University Innsbruck, Innsbruck, Austria; 3 Institute of Neuroradiology, Ludwig Maximilian University, Munich, Germany; 4 Department of Neuroradiology, University Hospital Carl Gustav Carus, Dresden, Germany

**Keywords:** Ocular motility, Diplopia, double vision, Vertigo, Ocular motor nerve palsies, Subjective visual vertical

## Abstract

**Objectives:**

Acute diplopia is a diagnostic challenge for clinicians, in particular in the emergency department. The most common cause of acute diplopia are ocular motor nerve palsies (OMP). In this prospective study, we focused on identifying the most crucial signs and symptoms for differentiating between peripheral and central OMP.

**Methods:**

We prospectively evaluated 56 non-consecutive patients who presented at our emergency department with acute binocular diplopia (≤ 10 days). The patient history was taken using a standardized questionnaire and patients underwent a neurological, neuro-ophthalmological and neuro-otological examination, including measurement of the subjective visual vertical (SVV), Harms tangent screen test, and cranial MRI.

**Results:**

Forty-six out of 56 patients were diagnosed with an ocular motor cranial nerve palsy (OMP), 21 of peripheral and 23 of central origin; in two patients, the etiology remained unknown. The following features were different in peripheral and central OMP: (1) the presence of vertigo/dizziness was more frequent in central (43.5%) than in peripheral (9.5%) OMP. (2) Central ocular motor signs, such as saccadic smooth pursuit, additional internuclear ophthalmoplegia, skew deviation, and saccade palsies, were also found more frequently in the central than in the peripheral group (86.7% vs. 33.3%). (3) Further, a pathological SVV deviation by monocular testing of the non-affected eye was also more common in central (77.3%) than in peripheral OMP (38.9%). The presence of all three factors has a positive predictive value of 100% (CI 50–100%) for the presence of a central lesion.

**Conclusions:**

In acute diplopia due to central OMP, the most important accompanying symptom is vertigo/dizziness, and the most important clinical signs are central ocular motor disorders (which require examination of the non-paretic eye) and an SVV deviation in the non-paretic eye.

## Introduction

Acute diplopia (perception of two different images) accounts for 0.1% of the patients that present at an emergency department [1]. An ocular misalignment of more than 200 µm [2] can cause binocular diplopia [3].

The most common causes of acute binocular diplopia are acute third (CNIII), fourth (CNIV) and sixth (CVI) cranial nerve palsies [1, 4, 5] (ocular motor palsies: OMP). The ocular motor nuclei are located throughout the brainstem, from the midbrain (CNIII) to the pons (CNIV) and the ponto-medullary junction (CNVI). Within the brainstem they have a long (CNIII, CNVI) or short (CNIV) so-called fascicular part [6]. After exiting the brainstem heading towards the eye, they pass through critical structures, such as the cavernous sinus. Because of their complicated anatomy and vicinity to these structures, prompt diagnosis of the localization of the OMP lesion is both very difficult and very important, in particular to diagnose a brainstem stroke. The importance of a prompt diagnosis in the emergency department is shown in a large prospective study [7], where 16% of the 50,000 emergency department visits due to diplopia were due to a life-threatening underlying disease.

There have been several prospective and retrospective studies on the etiologies of acute OMPs, each from a different point of view [5, 8–13]. The underlying diseases range from microvascular to acute life-threatening diseases, although the reported frequencies vary greatly from study to study.

In addition, Dieterich and Brandt have previously shown the important role of measuring mono- and binocular subjective visual verticality in the differentiation between a central and a peripheral lesion [14, 15].

In this study, we focus on the clinical differentiation of peripheral versus central OMPs in the acute phase in an effort to identify signs and symptoms that would assist a non-specialist in the emergency department in making the correct clinical decision regarding further diagnostic procedures and management.

## Methods

Over a period of 3 years, we prospectively recruited adult (≥ 18 years) patients from the neurological emergency department of Campus Grosshadern of the Ludwig Maximilian University, Munich, who presented because of acute diplopia. Diplopia had to be present for less than 10 days and be the leading symptom and the main cause for the patient’s referral to our department. Patients with monocular diplopia or binocular diplopia due to known pre-existing causes, such as myasthenia, were excluded from the study.

At the time of presentation, all patients underwent a complete clinical–neurological evaluation and had to answer a study questionnaire regarding their symptoms and presentation of diplopia. Furthermore, blood samples were obtained. A standardized neuro-ophthalmological and neuro-orthoptic examination, including Harms tangent screen [16] and monocular (each eye separately) measurements of the subjective visual vertical (SVV) [14, 15, 17], was performed on the same or the following day. The SVV was measured using either the bucket test or a dome. The SVV test was performed by an experienced orthoptician, who rotated the bucket manually and slowly from a randomized offset position towards zero, alternating between the two directions in each trial. The patient looked with one eye (the other eye was covered with an eye patch) at a straight line visible on the inner bottom of a bucket or of a dome without other visual cues, and had to align it properly, indicating the vertical position verbally. On the outer bottom surface of the bucket, an angular protractor allowed the examiner to readout the tilt angle; when the dome was used, the tilt angle was computed automatically [17]. Central ocular motor signs included saccadic smooth pursuit, saccade palsy, internuclear ophthalmoplegia, and skew deviation (in addition to the ocular motor palsy).

All patients but one (in whom the underlying diagnosis was evident in computed tomography) received an MRI (with DWI and MR angiography) 1–10 days after presentation. Lumbar puncture was performed when clinically indicated and when the cause of the OMP could not be detected in the MRI, meaning that it was performed in all patients with peripheral lesions, except for a patient with posttraumatic trochlear palsy. The final diagnosis was made taking into account all clinical, radiological and laboratory findings.

The patients were then divided into two categories according to their final diagnosis: peripheral or central OMP. Central OMP included both nuclear and fascicular lesions. The criteria for microvascular, peripheral OMP were based on the following criteria, reported earlier [18]: (1) isolated OMP (CNIII pupil sparing), (2) ESR less than 30 mm/hr and normal CRP, (3) age of 50 years or older, and (4) at least one of the cardiovascular risk factors: diabetes, hypertension, hyperlipidemia or smoking.

Fisher’s exact test, Chi-square test and Kruskal–Wallis test were used to compare the two groups. Statistical significance was set at *p* < 0.05. Statistical analysis was performed using IBM SPSS Statistics (Version 25.0.1), and calculation of sensitivity, specificity, positive and negative predictive value, and likelihood ratios was performed using vassarstats.net.

## Results

Fifty-six patients were initially recruited for the study. Ten of them were subsequently excluded after neuro-ophthalmological examination revealed another cause for the diplopia than an OMP (two patients with solely internuclear ophthalmoplegia, five patients with solely skew deviation, one patient with decompensated strabismus, one patient with thyroid disease and one patient with isolated inferior rectus muscle palsy due to IgG4-associated disease).

In the remaining 46 patients, a cause of the palsy could be identified in 44 patients. The two remaining patients had negative MRI and CSF findings and did not fulfill the criteria for any other disease (such as age and presence of cardiovascular risk factors), and were thus not included in the statistical evaluation.

The etiologies of the OMP are summarized in Table 1. Two patients with peripheral OMP (the patient with Fisher syndrome and the patient with idiopathic intracranial hypertension) and one with central OMP (glioma) had bilateral CNVI palsies.

**Table 1:** Etiologies of peripheral and central OMP

Peripheral		Central	
Etiology	#	Etiology	#
Ischemic	9	Ischemic	11
Infection	3	MS	7
Tumor	2	Tumor	3
Neurosarcoidosis	2	Vasculitis	1
Fisher syndrome	1	Cavernoma	1
Traumatic	1		
ACI dissection	1		
IIH	1		
OM	1		
Total	21		23

*ACI* internal carotid artery, *IIH* idiopathic intracranial hypertension, *OM* ophthalmoplegic migraine, *MS* multiple sclerosis

The results of the statistical comparison between the two groups are given in Table 2. The two groups did not differ statistically regarding age or gender. In both groups, CNVI was the most commonly affected, followed by CNIII and CNIV. Pupil involvement was documented in two out of nine patients with peripheral CNIII lesions (one with neuroborreliosis and one with sarcoidosis) and in two out of five patients with central lesions (both strokes).

**Table 2:** Clinical characteristics of the peripheral and central OMP group*s*

	Peripheral	Central	Total	*p* value
# Patients	21 (47.7%)	23 (52.3%)	44	
Ocular motor cranial nerve palsies	23 (48.9%)	24 (51.1%)	47	
CNIII	9 (39.1%)	5 (20.8%)	14 (29.8%)	0.26
CNIV	4 (17.4%)	3 (12.5%)	7 (14.9%)	
CNVI	10 (43.5%)	16 (66.7%)	26 (55.3%)	
Age ± SD (years)	58.8 ± 14.6	58.9 ± 17.2	58.8 ± 16.5	0.86^#^
Gender (women)	4 (19.0%)	11 (47.8%)	15 (34.1%)	0.06
First medical contact ± SD (days)	1.8 ± 0.9	2.1 ± 2.2	2.0 ± 2.2	0.46^#^
Neurological emergency ± SD (days)	2.9 ± 1.7	2.3 ± 2.2	2.5 ± 2.7	0.93^#^
Manifestation				
Acute	15 (71.4%)	16 (69.6%)	31 (70.5%)	1
Progressive	5 (23.8%)	5 (21.7%)	10 (22.7%)	
Woke up	1 (4.8%)	2 (8.7%)	3 (6.8%)	
Duration of symptoms				
Permanent	18 (85.7%)	19 (82.6%)	37 (84.1%)	0.20
Increasing	2 (9.5%)	0 (0%)	2 (4.5%)	
Fluctuating	1 (4.8%)	4 (17.4%)	5 (11.4%)	
Cardiovascular risk factors	1.6 ± 1.3	1.7 ± 1.4	1.6 ± 1.3	0.80^#^
Headache/periorbital pain	9 (42.9%)	6 (26.1%)	15 (34.1%)	0.34
Vertigo/dizziness	2 (9.5%)	10 (43.5%)	12 (27.3%)	**0.0174**
Other neurological symptoms	3 (14.3%)	4 (17.4%)	7 (15.9%)	1
Pathological SVV deviation paretic eye	10 (43.5%)	13 (54.2%)	23 (48.9%)	0.58
Pathological SVV deviation non-paretic eye	7 (38.9%)	17 (77.3%)	24 (60.0%)	**0.023**
Central ocular motor signs	7 (33.3%)	20 (86.7%)	27 (61.4%)	**0.0005**
Other neurological signs	7 (33.3%)	10 (43.5%)	17 (38.6%)	0.55

Bold indicates statistical significance

*p* refers to Fisher’s exact test for 2 × 2 tables and Chi square for 3 × 2 tables, apart from # that refers to the Kruskal–Wallis test

Both patient groups had their first medical contact on average 2 days after diplopia onset and were seen in our department on the same day or a day later. 70% of the patients had acute onset diplopia, in 23% the diplopia was progressive, and 7% of patients woke up with the symptoms (no difference between groups).

Regarding patients’ symptoms, statistical analysis showed that more central OMP patients reported concomitant vertigo or dizziness, whereas headache and/or periorbital pain were reported equally in both groups. Furthermore, both groups did not differ in terms of the presence of other neurological symptoms (such as hypoesthesia and paresthesia).

Regarding the clinical and neuro-ophthalmological findings, 87% of patients with a central OMP had additional central ocular motor deficits (such as spontaneous nystagmus, saccadic smooth pursuit, saccadic palsy on the healthy eye, internuclear ophthalmoplegia or skew deviation that was not justified by the underlying palsy, e.g. in CNVI palsies). 77% of the non-paretic eyes of patients with central OMP showed a pathological > 2.5° deviation, which was significantly more than in peripheral OMP (Table 2). One non-paretic eye in one patient with peripheral OMP could not be tested, due to severe congenital amblyopia. The patient groups showed no difference regarding the presence of other neurological signs.

Based on these findings, we calculated the sensitivity, specificity, positive predictive value, and negative predictive value of these three signs and symptoms (vertigo/dizziness, pathological subjective visual vertical in the non-paretic eye—SVVnp—and central ocular motor disorders-Omd) for identifying a central lesion (Table 3).

**Table 3:** Diagnostic performance of central ocular motor disorder (Omd), SVV deviation in the non-paretic eye (SVVnp) and vertigo/dizziness (V/D) for detecting central OMP

	Sensitivity	Specificity	PPV	NPV	LR+	LR−
Central OMP with Omd	0.87 (0.37–0.67)	0.67 (0.43–0.84)	0.74 (0.53–0.88)	0.82 (0.56–0.95)	2.61 (1.39–4.87)	0.19 (0.06–0.59)
Central OMP with SVVnp	0.77 (0.54–0.91)	0.61 (0.36–0.81)	0.71 (0.49–0.87)	0.69 (0.41–0.88)	1.98 (1.07–3.70)	0.37 (0.16–0.86)
Central OMP with V/D	0.43 (0.24–0.65)	0.90 (0.68–0.98)	0.83 (0.51–0.97)	0.59 (0.40–0.76)	4.57 (1.13–18.5)	0.62 (0.43–0.90)
Central OMP with all three factors	0.35 (0.17–0.57)	1 (0.81–1)	1 (0.50–1)	0.58 (0.41–0.74)	Infinity	0.65 (0.48–0.88)

*PPV* positive predictive value, *NPV* negative predictive value, *LR+* positive likelihood ratio, *LR−* negative likelihood ratio

According to our data, the concomitant presence of all three factors has a 100% specificity (CI 81–100%) and 100% positive predictive value (CI 50–100%). Therefore, a patient with OMP that reports vertigo or dizziness and has pathological SVV and central ocular motor disorder (Omd) on the non-affected eye most likely has a brainstem lesion.

## Discussion

The major finding of this study is that a differentiation between a central and a peripheral OMP lesion can be made clinically on the basis of the combination of (a) the patient history (presence of vertigo/dizziness or not), (b) neuro-ophthalmological examination of the non-paretic eye (central ocular motor disorders or not), and (c) measurement of subjective visual verticality on the non-affected eye [14].

In both our groups, CNVI was the most common documented palsy, followed by CNIII and CNIV, which is in line with previous literature [5, 8–13]. Clinically, a central lesion can be assumed with certainty only if the nuclei are involved: CNIII nuclear lesions can cause ipsilateral ophthalmoplegia with upgaze palsy and bilateral ptosis, CNIV nuclear lesions are associated with an ipsilateral Horner’s syndrome, and CNVI nuclear lesions with ipsilateral horizontal gaze palsy [19]. Nevertheless, these nerves, especially CNIII and CNVI, have a significant fascicular course through the brainstem [6] that cannot in itself be clinically differentiated with the same certainty from purely peripheral lesions.

Separating a brainstem stroke from a microvascular lesion is important for further therapeutic decisions; although secondary prophylaxis is mandatory in stroke, there is no evidence for or against the use of aspirin or oral anticoagulation in microvascular lesions. For example, in a retrospective study, aspirin did not have a protective effect against microvascular OMPs [20].

Although the presence of central ocular motor signs seems at first self-evident in central lesions, examination of the non-paretic eye is in our experience often neglected in clinical routine. Although most studies and reviews studying acute diplopia due to OMP concentrate on the clinical features of the paretic eye, our study further emphasizes the importance of a systematic examination of the central ocular motor system (saccades, smooth pursuit, cover test to unmask a skew deviation) in the non-paretic eye to detect a brainstem lesion [21]. The centers for ocular motor and vestibular control are abundant throughout the brainstem [22] and adjacent to the ocular motor nerve nuclei and fascicles. Therefore, a central brainstem lesion that affects them is also very likely to cause further central ocular motor deficits. Over the last years, clinical examination of the ocular motor system has been shown to be superior to MRI in identifying brainstem lesions [23–25], especially because ischemic brainstem lesions are often only detectable in the DWI more than 72 h after symptom onset [26]. Central ocular motor signs on the non-paretic eye are nevertheless not sufficient in themselves, since in our peripheral group 33.3% of all patients had central ocular motor signs (mostly saccadic smooth pursuit), which could be pre-existent in many of them.

A significant SVV deviation during binocular testing is considered to be a sign of dysfunction in the graviceptive pathways [27]. The importance of monocular) SVV testing for the differentiation of acute brainstem lesions from peripheral ocular motor palsies was already described before [14, 15]. In a retrospective study [14], about 40% of peripheral OMP showed a pathological SVV deviation in the affected eye during monocular testing, which was attributed to changes in visual perception through a lack of afferent extra-retinal information that codes the eye position in space. A pathological SVV in the non-paretic eye was previously documented only in chronic peripheral OMP [14] as a sign of central compensation for the contralateral palsy or in acute vascular brainstem lesions, representing an additional vestibular deficit in the roll plane [15].

The current work prospectively compares directly monocular SVV testing in acute peripheral versus central OMP. Monocular SVV deviation (> 2.5°) was observed in about 50% of all paretic eyes, independent of the localization of the lesion and the type of palsy. Regarding the non-paretic eye, pathologic SVV deviation was found in 77% of central lesions. This could be attributed to concomitant lesions in the graviceptive vestibular pathways that are anatomically adjacent to the ocular motor nuclei and fascicles [28]. In accordance with this, vertigo/dizziness were also reported more often in central than in peripheral OMP in our study. The patients with peripheral palsies described the sensation as dizziness. In the central group, one patient described a rotational vertigo sensation and two a falling tendency to one side (one to the right and one to the left), the rest described a dizziness sensation as well.

Surprisingly, there was also a small percentage of patients with peripheral OMP that showed a pathological SVV deviation when tested monocularly while viewing with the non-affected eye. In these patients, the pathological SVV deviation could be attributed either to an early compensation (like in the chronic OMP lesions [14]) or to an additional subclinical peripheral lesion in the seemingly healthy eye caused by a systemic disease, e.g. infection or sarcoidosis. Therefore, pathological SVV deviation in the clinically unaffected eye would justify further diagnostic steps (e.g. MRI, lumbar puncture), even if the OMP itself is clearly peripheral. The bedside examination of the SVV can be easily done using the bucket test [17].

Although pain is often postulated to be a feature of microvascular, peripheral OMP [29], patients with central lesions reported pain and ipsilateral headache as often as patients with peripheral palsy, especially when the central palsies were associated with a brainstem mass. There were no significant differences in the character of pain between the two groups; especially in the peripheral group, the pain was often described as diffuse and not strictly periorbital in our view. These findings undermine the significance of pain as the most significant diagnostic factor for a purely peripheral OMP.

The presence of other neurological signs did not exclude a peripheral lesion. Our patients with peripheral palsies also reported other neurological signs, such as hypesthesia, usually when the underlying disease affected other peripheral nerves, such as in the patient with Fisher syndrome.

Pupil sparing in CNIII palsy is usually considered a peripheral sign. In our study though, three out of five patients with central OMP did not have pupil involvement, which can be the case when the lesion is fascicular [3, 30]. On the other hand, two out of nine peripheral patients with CNIII palsy (due to sarcoidosis and neuroborreliosis) had pupil involvement.

**Fig. 1:**
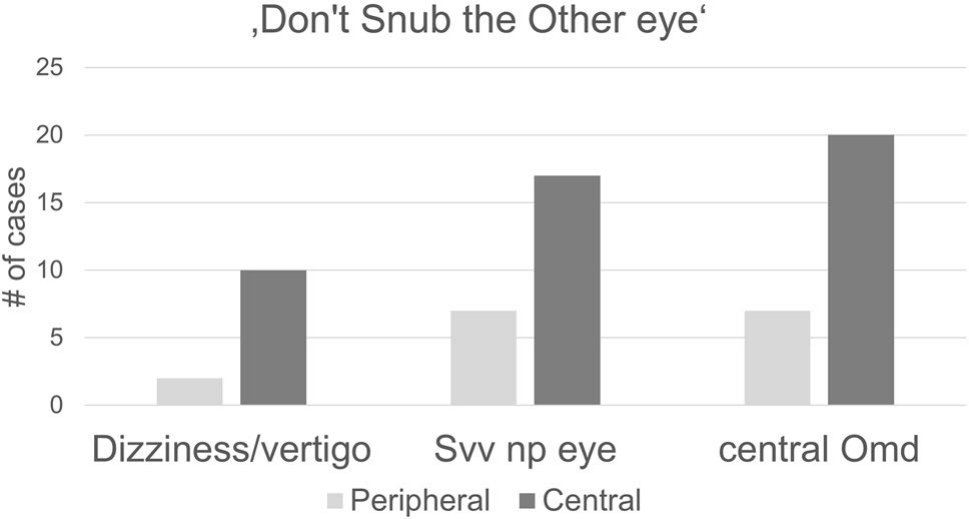
Number of positive cases of Dizziness/vertigo, SVV deviation and central Ocular motor disorder (Omd) in the non-paretic eye. The initials (DSO) can be remembered using the mnemonic device “Don’t Snub the Other eye”

The main findings are summed up in Fig. 1. Based on our data, we suggest the phrase (D)on’t (S)nub the (O)ther Eye (Dizziness, SVV and Ocular motor disorder of the non-paretic eye) as a mnemonic device for remembering these three factors that can assist the clinician in differentiating between acute peripheral and central OMP.

## Data Availability

The anonymized data can be shared by request from any qualified investigator for purposes of replicating procedures and results.
